# At the intersections: operationalizing intersectional thematic analysis in HIV prevention

**DOI:** 10.11606/s1518-8787.2024058005728

**Published:** 2024-10-11

**Authors:** Cristiane Spadacio, Lorruan Alves dos Santos, Ramiro Andres Fernandez Unsain, Isa da Silva Sorrentino, Marcia Thereza Couto

**Affiliations:** I Universidade de São Paulo. Faculdade de Medicina. Departamento de Medicina Preventiva. São Paulo, SP, Brasil Universidade de São Paulo Faculdade de Medicina Departamento de Medicina Preventiva São Paulo SP Brasil; II Universidade Católica de Santos. Programa de Pós-Graduação em Saúde Coletiva. Santos, SP, Brasil Universidade Católica de Santos Programa de Pós-Graduação em Saúde Coletiva Santos SP Brasil

**Keywords:** HIV, Intersectional Framework, Qualitative Research, Methods

## Abstract

**OBJECTIVE:**

This study aims to provide theoretical and methodological tools to assist in producing thematic analyses guided by an intersectional approach in empirically-based qualitative health studies. It argues that combining an intersectional perspective with thematic analysis can update the latter-which is quite popular in qualitative health investigations-regarding meaningful discussions about multiple and interconnected patterns of privilege and oppression that operate structurally and institutionally, producing experiences of relative disadvantage in individuals according to their gender, race/ethnicity, class, sexuality, generation, among other positions.

**METHODS:**

Based on an article that analyzed qualitative empirical data from a longitudinal demonstrative study on pre-exposure prophylaxis for HIV (PrEP) in adolescents and young people aged 15 to 19 years in two Brazilian capitals, this study discusses the limitations, challenges, and potentialities of the theoretical and methodological efforts undertaken by those authors. Additionally, this research that offers a proposal for operationalizing thematic analysis with intersectional sensitivity.

**RESULTS:**

It observed that triangulating techniques can enhance thematic analysis with intersectional sensitivity to produce qualitative data. Adopting an a priori intersectional proposal, starting from the research design phase, construction, and application of data production instruments with intersectional intentionality, enables the recognition of the relations between social markers in analytical categories.

**DISCUSSION:**

However, the absence of an intersectional theoretical-methodological perspective to conceive research and produce data fails to render intersectionality as a methodological tool unfeasible, although it may limit result analysis and discussion. Such limitations can be addressed by proposing intersectional assumptions and comparing the results with literature related to the theme and object of study.

## INTRODUCTION

Two analytical frameworks have been widely used in empirical health studies with a qualitative approach: content analysis and thematic analysis^1^^), (^^2^. Content analysis is a data analysis technique that originated in the Social Sciences in the 1950s and began to be widely used in the health field at the end of the twentieth century in both quantitative and qualitative research. It refers to a systematic coding and categorization process to find trends, patterns, and (inter)relations^3^. Thematic analysis, systematized by Braun and Clarke^4^, is also widely used. From this perspective, the theme evidences something representative of the research findings, presenting a pattern of responses. Due to its clearly defined character and established steps, this type of analysis has become very popular in qualitative investigations in the health field. Despite its popularization, thematic analysis has been used merely as a reference in the method section, with low fidelity to the technique application^5^.

Intersectionality, in turn, has been increasingly incorporated into health studies^6^^), (^^7^^), (^^8^. With its origins in American critical feminist studies of race and gender in the 1980s and 1990s^9^, the intersectional perspective question and act on the dynamics and complexity of social exclusion processes^10^. From a theoretical-political perspective, intersectionality reflects on themes, objects, productions, and scientific communications, as well as on researchers’ academic and political engagement^11^.

In methodological terms, intersectional analyses seek to explore the multiple and related patterns of privilege and oppression that operate at the macro social or structural level, impregnated in institutions, and how they produce experiences of relative disadvantage, oppressing individuals at the microsocial level according to their positions of gender, race/ethnicity, class, sexuality, generation, among others^11^^), (^^12^, which are conventionally called social categories of differentiation or social markers of difference.

The characteristics of academic publications with an intersectional approach hitherto produced in the Public Health field have revealed that intersectional studies find greater epistemological affinity with qualitative methodological approaches^13^. rises from the concern of qualitative research to shed light on the perceptions, representations, and meanings of the experiences of individuals socio-historically situated within the phenomena under analysis^6^.

Qualitative studies with an intersectional framework in the health field are increasing, and there is a great diversity of designs and techniques for producing qualitative data, including ethnography, case studies, interview techniques, focus groups, and field diaries, among others^14^. In addition, it is possible to verify that most of them, produced in the Global North or the Global South, aim to report inequalities and inequities, especially those related to access to health services and treatments^7^^), (^^15^^), (^^16^^), (^^17^^), (^^18^.

The field of HIV research has shown itself to be open to an intersectional perspective, both in epidemiological and qualitative studies. In epidemiological studies, this can be explained by how the contributions of vulnerability have developed in the field, including references to gender, sexuality, and race/ethnicity, as well as the need for references that would provide crossover opportunities between such categories^19^. In qualitative studies, the inclusion of other categories of social differentiation, in addition to sexuality, has been gradually incorporated, primarily due to the influence of feminist approaches. By engaging relational assumptions of gender with other social categories, they contributed not only to a broader and more dense understanding of the sociocultural dimensions of HIV/AIDS-such as stigma, social support, and the meaning of a life with HIV-but they also broadened the understanding of barriers to prevention and treatment access^20^^), (^^21^^), (^^22^. In both types of studies, the broad role of social movements in field struggles aligns with the political dimension of intersectionality as a resource for demanding rights and social justice^23^.

From our experience in HIV prevention with a qualitative approach and by operationalizing thematic analysis with intersectional sensitivity, the guiding question of this article emerges: is it possible to build a qualitative methodological framework anchored in thematic analysis with intersectional sensitivity? Therefore, the objective is to explore the potentialities and challenges of crossing over thematic analysis with intersectionality, based on a case of qualitative intersectional analysis produced in the field of HIV prevention, with adolescents from 15 to 19 years of age who use HIV Pre-Exposure Prophylaxis (PrEP)^24^.

## METHODS

This study has an essayistic and methodological character. The presented reflections are based on the propositions of thematic analysis and of intersectionality n qualitative research. They focus on an article that analyzed qualitative empirical data from a longitudinal study demonstrating PrEP among adolescents and young people aged 15 to 19 years in three Brazilian capitals, the *PrEP1519* study^25^.

The analyzed case was titled “PrEP perception and experiences of adolescent and young gay and bisexual men: an intersectional analysis”. It explored the perceptions and experiences of young gay, bisexual, and other men who have sex with men (GBMSM) in seeking, using, and adhering to PrEP, considering intersections of the social markers of difference regarding race/ethnicity, gender, class, sexuality, and generation, and how they operate as barriers and facilitators to the *continuum* of care in PrEP^24^.

That article was chosen as the studied case because of its analytical potential, which is part of the field intersection of qualitative health research, and by its analytical framework anchored in the thematic analysis of intersectional sensitivity based on an empirical study on the theme of HIV prevention. In addition, the case and this article are authored by members of the same research group.

The limitations, challenges, and potentialities of the theoretical and methodological efforts undertaken will be discussed, to propose a thematic analysis with intersectional sensitivity.

## RESULTS

The *PrEP1519* project is a longitudinal study with qualitative and quantitative components, conducted from February 2019 to September 2021, to evaluate the effectiveness of daily oral PrEP among adolescents who are men who have sex with men (MSM) and *travesty* and transsexual women (TrTW) from 15 to 19 years of age, in three Brazilian capitals: Belo Horizonte, Salvador, and São Paulo^25^.

The participants of the research, which was carried out with semi-structured interviews, were recruited using various strategies (via community interventions, dissemination on social networks, referrals in the health network, schools, and by the participants themselves). All of them joined the study after multidisciplinary clinical evaluation and testing, and they could opt for prevention combined with the use of PrEP (PrEP component) or without its use (Non-PrEP component).

In the qualitative component, the proposal to explore the sociability contexts of adolescents’ GBMSM and TrTW, their identity, and life contexts, including paying attention to situations of violence and oppression, provided the opportunity to elaborate scripts sensitive to intersectional data production^24^^), (^^26^.

As a methodological basis, this proposal takes an innovative approach, drawing from the perspectives of Abrams et al.^14^ and Hancock^27^. It underscores the importance of analyzing multiple categories of social differentiation without establishing a hierarchy and with a focus on the potential correlations expressed by the empirical data. This approach recognizes that the intersections between social categories are not a simple sum, but a complex interplay influenced by individual, institutional, and structural factors.

The case study considered 35 out of 55 semi-structured interviews with young gay, bisexual, and other men who have sex with men conducted in the qualitative component of *PrEP1519*. For an intersectional analysis, the selection needed to ensure the quality of the material-in terms of complete information on the subjects’ positions, according to the categories of social differentiation and density of narratives. Of the three steps of thematic analysis with an intersectional orientation established by Santos et al.^24^, we will analyze the third step, which, according to the authors, seeks to produce a synthesis based on the categories of social differentiation (gender identity, sexuality, race/ethnicity, generation, social condition) and their interrelations.

Regarding the methodological choice for an intersectional proposal, using semi-structured interviews and thematic analysis, it is possible to highlight the potential of correlating the thematic categories with the categories of social differentiation, perceiving in the course of the study which of the latter stood out in the participants’ speeches within the thematic categories. In the analyzed case, it is possible to show that some results highlight categories of social differentiation intersecting with some themes. In contrast, others do not show intersected categories of social differentiation.

**Chart 1:** Case study results with presence and absence of intersecting social markers.

Presence of intersecting social markers	
Themes	Social Markers
Economic risk	Sexuality
	Gender performativity
Repetitive risk situations	Sexuality
	Race/ethnicity
Repetitive risk situations	Social class
	Race/ethnicity
Initiation and adherence to PrEP use	Social class
	Race/ethnicity
Initiation and adherence to PrEP use	Sexuality
	Social class
	Race/ethnicity
**Absence of intersecting social markers**	
Family context	None
Network of family and friends	None
Use of condoms	None
Side Effects	None

Source: Authorial production based on the case study (Santos et al, 2023)

With the findings of articulated categories of social differentiation, we will present the intersections and the respective statements of the participants. On the position of the subject regarding “economic risk”, social categories such as sexuality and gender performativity intersect, as evidenced in the following case:

Regis, a São Paulo resident, who self-identifies as Black and gay, says: “I don’t think I’ve ever noticed because I’m Black, but because I’m more fem [effeminate], it’s harder. Let’s suppose I have AIDS, and a straight man has AIDS. I think that being a gay man with HIV carries more weight than if you’re a straight man”.

In the context of “repetitive risk”, the intersection of sexuality, class, race/ethnicity is evident:

Mauricio, who self-identifies as Black, gay, and non-binary and lives in Salvador, says that his Blackness works against him: “...being Black is already very hard... being Black and gay is even worse”.

And regarding the intersection of social class and race/ethnicity:

Vinicius (a gay, white resident of São Paulo), who self-identifies as being from a disadvantaged socioeconomic context, says: “I’m lower middle class, right?” However, by being White, he establishes socialization strategies: “So I have always been able to go to places that my social class would not normally allow me”.

In terms of the “initiation and adherence to PrEP use”, sexuality, class, and race/ethnicity also intersect:

Enrique (São Paulo resident), who self-identifies as gay and Black, defines his economic situation as “complicated” because he “depends on somebody else’s money” to live.

And categories such as sexuality, social class, and race/ethnicity:

Marcio (São Paulo resident) describes himself as “poor, ruined, really screwed”. Transportation costs make it difficult for him, but he tries to “get by” because “... I’m committed to this medication”. By identifying as Black, he recognizes the demand for “...a huge process of resignification”. As well as being Black and “poor”, being gay is “to be, above all, strong, in a homophobic, sexist, racist society. Being queer, Black, suburban, and an artist is an act of courage. It’s a daily challenge”.

For the set of cross-sectional findings, in which no category of specific social differentiation or its articulation stands out, we detected “family context” as an aspect that rejects non-hegemonic sexual behaviors, which results in a potentially unfavorable environment for the use of PrEP, with issues such as lack of privacy and family suspicion. Still, regarding the relational dimension, “family and friends” play a prominent role in adherence to PrEP, in the sense that participants do not need to hide the medication use and can count on a trusted support network, impacting the continuity and effectiveness of prophylaxis. There is also no apparent evidence of markers that determine “condom use”. Finally, the “side effects” of PrEP are presented as necessary in the daily prophylaxis, but they are also unrelated to specific social markers.

Based on the case analysis and theoretical-methodological discussions on the development and operationalization of intersectionality in qualitative empirical studies, an adaptation of the thematic analysis from an intersectional sensitivity lens is proposed (Chart 2).

**Chart 2:** Stages of thematic analysis and proposal for thematic analysis with intersectional sensitivity.

Stage	Thematic analysis^4^	Thematic analysis with intersectional sensitivity
**1**	**Pre-Analysis**	**Pre-Analysis/Contextualization**
	Definition of the analysis´s objectives and formulation of provisional hypotheses.	The definition of the analysis objectives and formulation of provisional hypotheses must consider the social and political contexts of the research theme and object.
	The selection and preparation of the empirical material are to be analyzed according to the research objective.	Selection and preparation of empirical material: reflexivity on the production phase of empirical data should inform the potentialities and limitations of each material, considering the proposal for articulating categories of social differentiation.
	Exhaustive reading of the material to be analyzed/impregnation.	Exhaustive reading of the material to be analyzed/impregnation with emphasis on the subjects’ positions according to the categories of social differentiation.
		The moment to identify which categories of social differentiation are present in the material and how they are expressed.
**2**	**Exploring the material**	**Exploring the material/Highlighting the social markers**
	Definition of the analysis units (themes/categories).	Definition of the analysis units (themes/categories).
	Encoding/extraction of contents (themes/categories).	Coding/extraction of contents (theme/categories) with the identification of the categories of social differentiation (if) present.
**3**	**Treatment of the obtained results and interpretation**	**Treatment of the obtained results and interpretation/Highlighting themes with (or without) associated social markers**
	Production of syntheses for each theme/category, evidencing the set of present meanings	Production of a synthesis for each theme/category, evidencing the set of present meanings, with identification of the presence (or not) of the intersecting categories of social differentiation.
	Interpretation regarding the proposed objectives, the literature on the theme/object, and the theoretical framework(s).	Interpretation regarding the proposed objectives, the literature on the theme/object and the theoretical intersectionality framework. In the final synthesis, the articulation of the categories of social differentiation and the way they operate in terms of the production of disadvantages/oppression and privileges should be highlighted.

## DISCUSSION

In the mentioned case, the crossing of the categories of social differentiation analyzed within the outlined themes/categories in the thematic analysis process allowed us to identify how structural aspects related to social class, gender, sexuality, and race/ethnicity are articulated in the production of different experiences and perceptions of disadvantages, but also of privilege, of adolescents and young people GBMSM about HIV prevention and the PrEP *continuum*.

Intersectional analyses should focus on critiques of social inequalities and reflect on essential justice policies. Therefore, critical self-reflexivity becomes fundamental throughout the process, as it is defended in qualitative research, whether about the researcher’s position, the political and social context of the research field and the data production, or the form of results analysis and dissemination^28^. Inseparably, intersectional analysis is enhanced by the principle of considering and involving research participants and the team throughout the process, to address and disclose divergences^29^. This aspect was not made explicit in the case. Still, we highlight the reference to the research team diversity in all cities regarding race/ethnicity, sexual orientation, and gender, emphasizing its importance in producing data sensitive to the investigation context^24^.

Since the data were produced to explain the experiences and tthe complexity of the perception according to the subjects’ positions regarding social markers, a fundamental component of stage 1 (see Chart 2), they needed to be analyzed intersectionally. However, even if the *a priori* presence of social markers of difference is considered in the elaboration of the research design, it does not mean *per se* that these markers will appear in the results. Even if the researcher does not directly ask participants about the social markers of difference and how they affect them, or if the participants make no explicit mention of them, the data should be examined in terms of how different social markers can shape particular experiences of oppression or privilege^30^. Therefore, there is no intersectional analysis without intersectional questions, as demonstrated in step 3 (Chart 2). In other words, empirical data never take the initiative since they answer what is asked^31^.

The analyzed case also offers clues to understand that the probable and predicted presence of categories of social differentiation in the investigation methodological design, or the emphasis given to them in the analysis, does not necessarily imply that all categories of social differentiation present the same weight in the observed social dynamics, neither means that they similarly express themselves in the analyzed thematic categories. In this case, it is clear that the categories of sexuality/gender, along with class, have a greater weight than, for example, the category of generation. On the other hand, if we analyze each interlocutor’s self-identification regarding race/ethnicity, we can observe that this category of social differentiation has different dynamics from the others according to the emic subjectivities. Thus, race/ethnicity emerges as a category of social differentiation that acts more irregularly than others (e.g., gender and sexuality).

In this sense, being able to perceive the intersections in the dynamics described and subsequently analyzed without being able to ponder the weights presented by each category of social differentiation could be a limiting aspect for a “complete” intersectional analysis. However, as Hill Collins and Bilge^12^ rightly argue, categories of social differentiation do not act separately but are in constant dialogue. Therefore, if these interactions are imbricated in a complex system of oppression/privilege, a plausible conclusion is that, in different contexts and temporalities, each of these categories of social differentiation is expressed with different weights. Or, as Cho et al.^32^ argue, what makes an analysis intersectional is the intention and action of handling the categories of social differentiation not as distinct or static entities but as mutually constructed and fluid, molded and continuously changing according to the power dynamics.

Another aspect that deserves to be highlighted is the spatio-temporal dimension of the analysis with intersectional sensitivity. The fields in which interview actions occur form “spaces of exception”^33^ and, undoubtedly, of reflection, which extend both in temporal and spatial terms. That is why, from a qualitative perspective, and particularly from an intersectional sensibility perspective, the field not only materializes regarding the specific area to be researched but also the subjects who will become interlocutors. This field will also be the entire universe of meanings recreated outside these spaces and times.

During the production of our case, it was also possible to observe that analysis with an a priori intersectional intentionality favors and enables the recognition of the relations between categories of social differentiation and themes/categories of analysis. However, the fact that the theoretical-methodological perspective of intersectionality is absent from the conception of the research and in data production does not preclude this framework use, although it may imply some limitations to the analysis and discussion of the results^30^. To account for this limitation, it is possible to consider hypotheses/theses from the literature to support the analysis. As stated by Bowleg et al.^34^, theory is necessary when opening up to the complex and interconnected data analysis and interpretation according to the framework of intersectionality.

As we have argued, the intersectional perspective is a framework committed to an epistemological perspective based on political and academic engagement to promote social transformations^12^^), (^^35^. We claim that the intersectional perspective in dialogue with thematic analysis should not ignore the political, economic, and social context of research and knowledge production. However, we should not forget the crucial reflection of Bowleg et al.^34^ on the fact that qualitative methodologies should not be considered progressive *a priori*: they will only be so if they are committed to countering the so-called epistemologies of ignorance, which intentionally cover up certain aspects of reality to justify and reiterate the *status quo*.

## CONCLUSION

Although researchers in the health field are enthusiastic about developing intersectional analyses in qualitative studies and are increasingly pursuing this approach, the substantive use of intersectional methodological assumptions still needs to be improved. We proposed to explain the values existing in knowledge production according to an intersectional reference and critically evaluating how they operated within a case. Next, we sought to present a proposal to operationalize intersectional thematic analysis to expand the possibilities for the intersectionality methodological framework.

When referring to thematic analysis and the theoretical-methodological-political perspective regarding intersectionality, a distinction between methodology/analysis technique is considered. Methodology/technique of analysis is understood as concrete processes that researchers will face and develop step by step for the construction of the analysis. From the theoretical-methodological-political perspective, intersectionality is a “lens of analysis” structured in three axes: reflexivity, the non-prioritization of categories of social differentiation a priori, and the need to look at the categories of social differentiation in an articulated way, not as a mere summation.

We understand that although methodological debates regarding the operationalization of analysis proposals are fundamental, they will be innocuous and premature if they lack discussions anchored in epistemological assumptions. Intersectional analyses are rigorous and require theory at multiple levels to reveal theoretical connections that are often only implicit^29^^), (^^30^^), (^^35^.

Perhaps the most fruitful use of intersectionality is an analytical strategy or a willingness to investigate and produce new knowledge about social phenomena^36^. Thus, the attempt to reflexively approach the intersectional perspective of one of the most used data analysis techniques in the health field, especially in the Brazilian context, with thematic analysis. Such processes are conducted to enable innovative and expanded analyses that are sensitive to the social markers of difference, problematize their impacts in research contexts, and reaffirm their necessary implication with social justice and equity in health.
