# Errata

**DOI:** 10.11606/s1518-8787.2025059005728err

**Published:** 2025-06-27

No artigo “Nas intersecções: operacionalizando análise temática interseccional em prevenção ao HIV”, DOI http://doi.org/10.11606/s1518-8787.2020054005728, publicado na Revista de Saúde Pública. 2024;58: Suppl 1:5s, RSP corrige:

**Como citar (página 1):**

Onde se lê:

http://doi.org/10.11606/s1518-8787.2020054005728

Leia-se:

http://doi.org/10.11606/s1518-8787.2024058005728

**Rodapé (páginas 1 a 10):**

Onde se lê:

http://doi.org/10.11606/s1518-8787.2020054005728

Leia-se:

http://doi.org/10.11606/s1518-8787.2024058005728

