# Supplementary material for: At the intersections: operationalizing intersectional thematic analysis in HIV prevention
Source: Rev Saude Publica. 2024 Oct 11;58(Suppl 1):5s. doi: 10.11606/s1518-8787.2024058005728 (PMC11573374; doi:10.11606/s1518-8787.2024058005728)
Supplement: Translation

# Translation: Nas intersecções: operacionalizando análise temática interseccional em prevenção ao HIV

**Authors:** Cristiane Spadacio, Lorruan Alves dos Santos, Ramiro Andres Fernandez Unsain, Isa da Silva Sorrentino, Marcia Thereza Couto

**Affiliations:** I Universidade de São Paulo. Faculdade de Medicina. Departamento de Medicina Preventiva. São Paulo, SP, Brasil Universidade de São Paulo Faculdade de Medicina Departamento de Medicina Preventiva São Paulo SP Brasil; II Universidade Católica de Santos. Programa de Pós-Graduação em Saúde Coletiva. Santos, SP, Brasil Universidade Católica de Santos Programa de Pós-Graduação em Saúde Coletiva Santos SP Brasil

**Keywords:** HIV, Enquadramento Interseccional, Pesquisa Qualitativa, Métodos

## Abstract

**OBJETIVO:**

Este artigo objetiva fornecer instrumentação teórica e metodológica inicial para auxiliar a produção de análises temáticas sob orientação interseccional em estudos qualitativos de base empírica em saúde. Argumenta-se que o encontro da perspectiva interseccional com a análise temática tem o potencial de atualizar essa última - bastante popular nas investigações qualitativas no campo da saúde - acerca das importantes discussões sobre os múltiplos e relacionados padrões de privilégio e opressão que operam nos níveis estrutural e institucional e produzem experiências de desvantagem relativa nos indivíduos segundo suas posições de gênero, raça/cor, classe, sexualidade, geração, entre outras.

**MÉTODO:**

Este trabalho, com caráter ensaístico e metodológico, foi realizado a partir de um artigo que analisou dados empíricos qualitativos de um estudo longitudinal demonstrativo da Profilaxia Pré-Exposição sexual ao HIV (PrEP) entre jovens de 15 a 19 anos em três capitais brasileiras. Foram discutidas as limitações, os desafios e as potencialidades dos esforços teóricos e metodológicos empreendidos pelos seus autores, e apresentada uma proposta de operacionalização da análise temática com sensibilidade interseccional.

**RESULTADOS:**

Observou-se que a análise temática com sensibilidade interseccional pode ser potencializada com a utilização de triangulação de técnicas para a produção de dados qualitativos. A proposição inicial, desde a etapa do desenho da pesquisa, da construção e da aplicação dos instrumentos de produção de dados com intencionalidade interseccional favorece e viabiliza o reconhecimento das relações entre marcadores sociais nas categorias analíticas.

**DISCUSSÃO:**

O fato de a perspectiva teórico-metodológica da interseccionalidade não estar presente desde a concepção da pesquisa e a fase de produção dos dados não inviabiliza a utilização da interseccionalidade como ferramenta metodológica, embora possa implicar algumas limitações em termos de análise e discussão dos resultados. Tais limitações podem ser contornadas com a proposição de pressupostos de caráter interseccional e o cotejamento dos resultados com a literatura referente ao tema e objeto de estudo.

## INTRODUÇÃO

Nos estudos empíricos em saúde com abordagem qualitativa, dois referenciais de análise têm sido amplamente utilizados: a análise de conteúdo e a análise temática^1^^), (^^2^. A análise de conteúdo é uma técnica de análise de dados oriunda das Ciências Sociais na década de 1950 e que, no final do século XX, passou a ser amplamente utilizada no campo da saúde, tanto em pesquisas quantitativas como em pesquisas qualitativas. Refere-se a um processo sistemático de codificação e categorização com o intuito de encontrar tendências, padrões e (inter)relações^3^. A análise temática, sistematizada por Braun e Clarke^4^, é também bastante utilizada. Nessa perspectiva, o tema evidencia algo representativo dos achados da pesquisa, apresentando um padrão das respostas. Por seu caráter claramente definido e com passos estabelecidos, esse tipo de análise se tornou bastante popular nas investigações qualitativas no campo da saúde. Não obstante sua popularização, a análise temática tem sido usada meramente como uma referência na seção de método, com baixa fidelidade à aplicação da técnica^5^.

A interseccionalidade, por sua vez, vem sendo incorporada cada vez mais nos estudos em saúde^6^^), (^^7^^), (^^8^. Originária dos estudos críticos feministas estadunidenses de raça e gênero nos anos 1980 e 1990^9^, a perspectiva interseccional é útil para interrogar e agir diante da dinâmica e complexidade dos processos de exclusão social^10^. Enquanto perspectiva teórico-política, a interseccionalidade adota a reflexividade sobre os temas, objetos, produções e comunicações científicas, bem como sobre o engajamento acadêmico e político dos pesquisadores^11^.

Em termos metodológicos, as análises interseccionais buscam explorar os múltiplos e relacionados padrões de privilégio e opressão que operam no nível macrossocial ou estrutural impregnados nas instituições e como estes produzem experiências de desvantagem relativa, oprimindo os indivíduos no nível microssocial segundo suas posições de gênero, raça/cor, classe, sexualidade, geração, entre outras^11^^), (^^12^, o que se convencionou chamar de categorias sociais de diferenciação ou marcadores sociais da diferença.

As características das publicações acadêmicas com abordagem interseccional produzidas até hoje no campo da Saúde Pública revelam que os estudos interseccionais encontram maior afinidade epistemológica com as abordagens metodológicas qualitativas^13^. Isso decorre da preocupação da pesquisa qualitativa em lançar luz sobre as percepções, as representações e os significados das experiências dos indivíduos sócio-historicamente situados acerca dos fenômenos em análise^6^.

Estudos qualitativos com referencial interseccional no campo da Saúde são crescentes, com grande diversidade de desenhos e técnicas de produção de dados qualitativos, incluindo etnografia, estudo de caso, assim como técnicas de entrevista, grupos focais e diários de campo, entre outros^14^. Além disso, é possível constatar que boa parte destes, produzidos no Norte Global ou no Sul Global, objetivam denunciar as desigualdades e iniquidades, especialmente relacionadas ao acesso aos serviços e tratamentos em saúde^7^^), (^^15^^), (^^16^^), (^^17^^), (^^18^.

O campo das pesquisas em HIV tem se mostrado aberto à perspectiva interseccional, tanto em estudos epidemiológicos quanto qualitativos. Nos estudos epidemiológicos, isso pode ser explicado pela maneira como as contribuições da vulnerabilidade se desenvolveram no campo, incluindo referências à gênero, sexualidade e raça/cor, bem como à necessidade de referenciais que oportunizassem o cruzamento entre essas categorias^19^. Nos estudos qualitativos, a inclusão de outras categorias de diferenciação social, além da sexualidade, foi paulatinamente incorporada, em grande parte devido à influência de abordagens feministas. Estas, ao acionarem pressupostos relacionais de gênero com outras categorias sociais, contribuíram não só para a compreensão alargada e adensada das dimensões socioculturais do HIV/aids - como o estigma, suporte social e o significado da experiência de viver com HIV -, mas também ampliaram a compreensão das barreiras de acesso à prevenção e tratamento^20^^), (^^21^^), (^^22^. Em ambos os tipos de estudo, a ampla atuação dos movimentos sociais nas lutas do campo vai ao encontro da dimensão política da interseccionalidade como recurso para a luta por direitos e justiça social^23^.

A partir da nossa experiência no campo de conhecimento da prevenção ao HIV com abordagem qualitativa, e no esforço produzido ao operacionalizar análise temática com sensibilidade interseccional, emerge a questão norteadora deste artigo: é possível construir um arcabouço metodológico qualitativo ancorado na análise temática com sensibilidade interseccional? Diante disso, objetiva-se explorar as potencialidades e desafios do entrecruzamento da análise temática com a interseccionalidade, a partir de um *case* de análise interseccional qualitativa produzido no campo da prevenção ao HIV, com adolescentes de 15 a 19 anos que usam a Profilaxia Pré-Exposição sexual ao HIV (PrEP)^24^.

## MÉTODOS

Este estudo tem caráter ensaístico e metodológico. As reflexões aqui apresentadas ancoram-se nas proposições da análise temática e da interseccionalidade em pesquisas qualitativas, com foco em um artigo que analisou dados empíricos qualitativos de um estudo longitudinal demonstrativo de PrEP entre adolescentes e jovens de 15 a 19 anos de três capitais brasileiras, o Estudo *PrEP1519*^25^.

O *case* em análise, intitulado *Percepção e experiências da PrEP de homens gays e bissexuais jovens e adolescentes: uma análise interseccional*, explorou as percepções e experiências de homens jovens gays, bissexuais e outros homens que fazem sexo com homens (GBHSH) na busca, uso e adesão à PrEP, considerando intersecções dos marcadores sociais da diferença de raça, gênero, classe, sexualidade e geração, e como estes operam barreiras e facilitadores para o *continuum* do cuidado em PrEP^24^.

A escolha desse artigo como *case* justifica-se pelo seu potencial analítico, que se insere no entrecruzamento do campo da pesquisa qualitativa em saúde, e utiliza um referencial analítico ancorado na análise temática de sensibilidade interseccional a partir de um estudo empírico no tema da prevenção ao HIV. Ademais, a produção do *case* e deste artigo é de autoria de integrantes do mesmo grupo de pesquisa.

Discutiremos as limitações, desafios e potencialidades dos esforços teóricos e metodológicos empreendidos, que possibilitam apresentar uma proposta de análise temática com sensibilidade interseccional.

## RESULTADOS

O projeto *PrEP1519* é um estudo longitudinal com componentes qualitativos e quantitativos, conduzido entre fevereiro de 2019 e setembro de 2021, para avaliar a efetividade de PrEP oral diária entre adolescentes HSH e Mulheres Transsexuais e Travestis (MTrT) com idade entre 15 e 19 anos, em três capitais brasileiras: Belo Horizonte, Salvador e São Paulo^25^.

Os(as) participantes da pesquisa, realizada com entrevistas semiestruturadas, foram recrutados(as) a partir de diversas estratégias de captação (por intervenções comunitárias, divulgação em redes sociais, encaminhamentos por meio da rede de saúde, de escolas e pelos(as) próprios(as) participantes). Todas(os) ingressaram no estudo após avaliação clínica multiprofissional e testagens, podendo optar pela prevenção combinada com o uso da PrEP (componente PrEP) ou sem o uso dela (componente Não-PrEP).

No componente qualitativo, a proposta de explorar os contextos de sociabilidade dos adolescentes BGHSH e MTrT, seus pertencimentos identitários e os contextos de vida, inclusive dando atenção às situações de violência e opressão, oportunizou a construção de roteiros sensíveis à produção de dados interseccionais^24^^), (^^26^.

Como fundamentação metodológica, o artigo *case* em análise se apoiou na perspectiva de Abrams et al.^14^ e Hancock^27^, que indica a necessidade de incluir na análise mais de uma categoria de diferenciação social em articulação, sem estabelecimento prévio de hierarquia entre elas e com destaque para as possíveis correlações expressas pelos dados empíricos. Assim, considerou-se que as intersecções entre as categorias de diferenciação social não representam a mera soma das categorias sociais existentes, e o peso destas deve levar em conta os contextos sociais envolvidos em sua (re)produção, o que leva em conta uma dinâmica que envolve fatores individuais, institucionais e estruturais.

O estudo *case* considerou 35 de 55 entrevistas semiestruturadas com jovens gays, bissexuais e outros homens que fazem sexo com homens realizadas no componente qualitativo do *PrEP1519*. A seleção se deveu à necessidade de garantir a qualidade do material - em termos de informações completas das posições dos sujeitos, segundo as categorias de diferenciação social e densidade de narrativas - para uma análise interseccional. Dos três passos de análise temática com orientação interseccional estabelecidos por Santos et al.^24^, analisaremos o terceiro passo, que, de acordo com os autores, busca produzir uma síntese baseada nas categorias de diferenciação social (identidade de gênero, sexualidade, raça/cor, geração, condição social) e nas suas inter-relações.

Sobre a escolha metodológica por uma proposta interseccional, com a utilização de entrevista semiestruturada e análise temática, é possível destacar a potencialidade de correlacionar as categorias temáticas com as categorias de diferenciação social, percebendo no decorrer da análise quais dessas últimas se sobressaíram nas falas dos participantes no interior das categorias temáticas. No *case* sob análise, é possível evidenciar que alguns resultados ressaltam categorias de diferenciação sociais intersectadas com alguns temas e outros não evidenciam categorias de diferenciação social intersectadas.

**Quadro 1:** Resultados do *case* com presença e ausência de marcadores sociais intersectados.

Presença de marcadores sociais intersectados	
Temas	Marcadores
Economia de risco	Sexualidade
	Performatividade de gênero
Situações de risco repetitivo	Sexualidade
	Raça/cor
Situações de risco repetitivo	Classe social
	Raça/cor
Início e adesão ao uso de PrEP	Classe social
	Raça/cor
Início e adesão ao uso de PrEP	Sexualidade
	Classe social
	Raça/cor
**Ausência de marcadores sociais intersectados**	
Contexto familiar	Não há
Rede de família e amigos	Não há
Uso de camisinha	Não há
Efeitos colaterais	Não há

Fonte: Confecção própria a partir do *Case* (Santos et al, 2023)

No conjunto de achados com categorias de diferenciação social articuladas, apresentaremos as intersecções e as respectivas falas dos participantes. Quanto à posição do sujeito na chamada “economia de risco”, categorias sociais como sexualidade e performatividade de gênero se intersectam, tal como evidenciado no *case*:

Regis, a São Paulo resident, who self-identifies as black and gay, says: “I don’t think I’ve ever noticed because I’m black, but because I’m more fem [afeminado], it’s harder. Let’s suppose I have AIDS and a straight man has AIDS. I think that being a gay man with HIV carries more weight than if you’re a straight man.

No contexto de “risco repetitivo”, evidencia-se a intersecção entre sexualidade, classe, raça/cor:

Mauricio, who self-identifies as black, gay and non-binary, and lives in Salvador, says that his blackness works against him: “...being black is already very hard... being black and gay is even worse”

E com a intersecção entre classe social e raça/cor:

Vinicius (a gay, white São Paulo resident), who self-identifies as being from a disadvantaged socioeconomic context: “I’m lower middle class, right?”. However, because he is white, he establishes socialization strategies: “So I have always been able to go to places that my social class would not normally allow me.

Em termos do “início da adesão e uso da PrEP”, sexualidade, classe e raça/cor também se intersectam:

Enrique (São Paulo), who self-identifies as gay, black defines his economic situation as “complicated” because he “depends on the money that’s not his” to live.

E categorias como sexualidade, classe social e raça/cor:

Marcio, (São Paulo) describes himself as “poor, ruined, really screwed”. Transport costs make it difficult for him, but he tries to “get by” because “...I’m committed to this medication”. By identifying as black, he recognizes the demand for “...a huge process of resignification”. As well to being black and “poor”, being gay is “to be, above all, strong, in a homophobic, sexist, racist society. Being queer, black, suburban, and an artist is an act of courage. It’s a daily challenge”

Para o conjunto de achados transversais, nos quais nenhuma categoria de diferenciação social específica ou articulação desta se destacam, detectamos que o “contexto familiar” se apresenta como um aspecto que rejeita comportamentos sexuais não hegemônicos, o que resulta em um ambiente potencialmente desfavorável para o uso da PrEP, com questões como falta de privacidade e suspeita familiar. Ainda em relação à dimensão relacional, “família e amigos” têm um papel de destaque quando se trata da adesão à PrEP, no sentido de os participantes não precisarem esconder o uso da medicação e poderem contar com rede de apoio de confiança, impactando na continuidade e eficácia da profilaxia. Também não parece haver evidências de marcadores que determinem o “uso da camisinha”. Finalmente, os “efeitos colaterais” da PrEP evidenciam-se como importantes no uso diário da profilaxia, porém também não estão relacionados a marcadores sociais específicos.

A partir da análise do *case*, e à luz de discussões teórico-metodológicas sobre o desenvolvimento e a operacionalização da interseccionalidade em estudos empíricos qualitativos, é proposta uma adaptação da análise temática a partir de uma lente de sensibilidade interseccional (Quadro 2).

**Quadro 2:** Etapas da análise temática e proposta de operacionalização da análise temática com sensibilidade interseccional.

Etapa	Análise temática^4^	Análise temática com sensibilidade interseccional
**1**	**Pré-Análise**	**Pré-Análise/Contextualização**
	Definição dos objetivos a serem alcançados pela análise e formulação de hipóteses provisórias.	A definição dos objetivos da análise e formulação de hipóteses provisórias devem levar em conta os contextos sociais e políticos do tema e objeto da pesquisa.
	Seleção e preparo do material empírico a ser analisado de acordo com o objetivo da pesquisa.	Seleção e preparo do material empírico: a reflexividade sobre a fase de produção dos dados empíricos deve informar as potencialidades e limitações de cada material a ser analisado considerando a proposta de articulação de categorias de diferenciação social.
	Leitura exaustiva do material a ser analisado/impregnação.	Leitura exaustiva do material a ser analisado/impregnação com destaque para as posições dos sujeitos segundo as categorias de diferenciação social.
		Momento de identificar quais categorias de diferenciação social estão presentes no conjunto do material e como se expressam.
**2**	**Explorando o material**	**Explorando o material/Evidenciando os marcadores sociais**
	Definição das unidades de análise (temas/categorias).	Definição das unidades de análise (temas/categorias)
	Codificação/extração dos conteúdos (temas/categorias).	Codificação/extração dos conteúdos (tema/categorias) com identificação das categorias de diferenciação social (quando) presentes.
**3**	**Tratamento dos resultados obtidos e interpretação**	**Tratamento dos resultados obtidos e interpretação/Evidenciando temas com (ou sem) marcadores sociais associados**
	Produção de sínteses para cada tema/categoria, evidenciando o conjunto de significados presentes	Produção de síntese para cada tema/categoria, evidenciando o conjunto dos significados presentes, com identificação da presença (ou não) das categorias de diferenciação social intersectadas.
	Interpretação à luz dos objetivos propostos, da literatura sobre o tema/objeto e do(s) referencial(is) teórico(s).	Interpretação à luz dos objetivos propostos, da literatura sobre o tema/objeto e do referencial teórico da interseccionalidade. Destaque, na síntese final, a articulação das categorias de diferenciação social e a forma como operam em termos da produção de desvantagens/opressão e privilégios.

## DISCUSSÃO

No *case* apresentado, o cruzamento das categorias de diferenciação sociais analisadas no interior dos temas/categorias delineadas no processo da análise temática permitiu identificar como aspectos estruturais relativos à classe social, gênero, sexualidade e raça/cor da pele se articulam na produção de diferentes experiências e percepções de desvantagens, mas também de privilégio, dos adolescentes e jovens GBHSH com relação à prevenção do HIV e ao *continuum* de PrEP.

Análises interseccionais devem estar atentas às críticas às desigualdades sociais e refletir sobre quais políticas de justiça são essenciais. Portanto, a autorreflexividade crítica, defendida na pesquisa qualitativa, seja quanto à posição do pesquisador, ao contexto político social do campo de pesquisa e da produção dos dados ou à forma de análise e divulgação dos resultados, torna-se fundamental em todo o processo^28^. De modo indissociável, o princípio de considerar e envolver os participantes da pesquisa e da equipe ao longo do processo, de forma a atender e divulgar divergências, certamente potencializa a análise interseccional^29^. Tal aspecto não foi explicitado no *case*, mas ressalta-se a referência à diversidade do time de pesquisadores, em todas as cidades, quanto à raça/cor, à orientação sexual e ao gênero, enfatizando sua importância na produção de dados sensíveis ao contexto de investigação^24^.

Uma vez que os dados foram produzidos de forma a explicitar a complexidade das experiências e percepções segundo as posições dos sujeitos relativas aos marcadores sociais, componente fundamental da etapa 1 (ver Quadro 2), eles precisaram ser analisados de forma interseccional. Todavia, ainda que a presença *a priori* de marcadores sociais da diferença seja considerada na elaboração do desenho da investigação, não significa *per se* que esses marcadores vão aparecer nos resultados. E mesmo que o pesquisador não pergunte diretamente aos participantes sobre os marcadores sociais da diferença e a forma como eles os atravessam, ou que os participantes não façam menção explícita a eles, os dados devem ser interrogados em termos de como diferentes marcadores sociais podem conformar experiências particulares de opressão ou privilégio^30^. Portanto, não há análise interseccional sem perguntas interseccionais, como demostrado na etapa 3 (Quadro 2). Em outras palavras: os dados empíricos nunca tomam iniciativa, já que dão resposta ao que é perguntado^31^.

O *case* analisado também oferece pistas para compreender que a presença provável e prevista de categorias de diferenciação social no desenho metodológico da investigação, ou a ênfase dada a elas no processo de análise, não implica necessariamente que todas as categorias de diferenciação social apresentem o mesmo peso nas dinâmicas sociais observadas e se expressem de forma semelhante nas categorias temáticas elegidas para análise. No *case*, é claro que as categorias sexualidade/gênero, junto com classe, apresentam um maior peso do que, por exemplo, a categoria geração. Por sua vez, se analisamos como cada interlocutor considera seu pertencimento em termos de raça/cor, podemos observar que há diferentes valorações em como essa categoria de diferenciação social dinamiza com as demais, segundo as subjetividades êmicas. Assim, raça/cor surge como uma categoria de diferenciação social que atua mais irregularmente do que outras (por exemplo, gênero e sexualidade).

Nesse sentido, conseguir perceber as intersecções presentes nas dinâmicas descritas e, posteriormente, analisadas, sem conseguir ponderar os pesos que cada categoria de diferenciação social apresenta, poderia ser um aspecto limitante para uma análise interseccional “completa”. Entretanto, como Hill Collins e Bilge^12^ bem argumentam, as categorias de diferenciação social não atuam separadamente, mas dialogam constantemente. Portanto, se essas interações estão imbricadas em um sistema complexo de opressão/privilégio, uma conclusão plausível é que, em contextos e temporalidades diferentes, cada uma dessas categorias de diferenciação social se expressa com diferentes pesos. Ou, como defende Cho et al.^32^, o que faz uma análise ser interseccional é a intenção e ação de manejar as categorias de diferenciação social não como entidades distintas ou estáticas, mas como mutuamente construídas e fluidas, moldadas e moldando-se continuamente segundo as dinâmicas do poder.

Outro aspecto merece destaque: a dimensão espaço-temporal da análise com sensibilidade interseccional. Os campos em que se desenrolam as ações das entrevistas conformam “espaços de exceção”^33^ e, sem dúvida, de reflexão, que se estendem tanto em termos temporais como espaciais. É por isso que, sob a perspectiva qualitativa, e particularmente a de sensibilidade interseccional, o campo não somente se materializa em relação à área específica a pesquisar e aos(às) sujeitos(as) que se tornarão interlocutores(as): esse campo também será todo o universo de sentidos recriado fora desses espaços e tempos.

Foi possível também observar, durante a produção do nosso *case*, que uma análise com intencionalidade interseccional *a priori* favorece e viabiliza claramente o reconhecimento das relações entre categorias de diferenciação social e categorias/temas de análise. Mas o fato de a perspectiva teórico-metodológica da interseccionalidade não estar presente desde a concepção da pesquisa e na fase de produção dos dados não inviabiliza a utilização desse referencial, embora possa implicar em algumas limitações em termos de análise e discussão dos resultados^30^. Para dar conta de tal limitação, é possível considerar hipóteses/teses advindas da literatura para subsidiar a análise. E, como afirma Bowleg et al.^34^, ao se abrir à complexa e interconectada análise e interpretação de dados segundo o referencial da interseccionalidade, a teoria é necessária.

Conforme argumentamos, a perspectiva interseccional é um referencial comprometido com uma perspectiva epistemológica pautada no engajamento político e acadêmico para promover transformações sociais^12^^), (^^35^. Sustentamos que a perspectiva interseccional em diálogo com a análise temática não deve ignorar o contexto político, econômico e social de pesquisa e produção de conhecimento. Todavia, não devemos esquecer a importante reflexão de Bowleg et al.^34^ sobre o fato de que as metodologias qualitativas não devem ser consideradas progressistas *a priori*: só o serão se estiverem comprometidas em contrapor as chamadas epistemologias da ignorância, que intencionalmente encobrem determinados aspectos da realidade a fim de justificar e reiterar o *status quo*.

## CONCLUSÕES

Embora o entusiasmo por desenvolver análises interseccionais em estudos qualitativos seja louvável e cada vez mais perseguido por pesquisadores no campo da saúde, a utilização de pressupostos metodológicos interseccionais de forma substantiva é ainda pouco efetiva. Propusemos explicitar o conjunto de valores existentes na produção de conhecimento segundo referencial interseccional e avaliar criticamente como estes operaram em um *case.* Na sequência, buscamos apresentar uma proposta de operacionalização de análise temática interseccional, de modo a expandir a gama de possibilidades de utilização do referencial metodológico da interseccionalidade.

Considera-se que há uma distinção entre metodologia/técnica de análise, quando nos referimos à análise temática, e perspectiva teórico-metodológica-política, quando se trata da interseccionalidade. Por metodologia/técnica de análise são entendidos processos concretos com os quais os pesquisadores irão se deparar e desenvolver o passo a passo para a construção da análise. Na perspectiva teórico-metodológica-política, a interseccionalidade se apresenta como uma “lente de análise” estruturada em três eixos: a reflexividade, a não priorização de categorias de diferenciação social *a priori* e a necessidade de olhar as categorias de diferenciação social de forma articulada, e não como uma mera somatória.

Entendemos que, embora os debates metodológicos em termos de operacionalização de propostas de análise sejam fundamentais, eles serão inócuos e prematuros se carecerem de discussões ancoradas em pressupostos epistemológicos. As análises interseccionais são rigorosas e requerem o uso da teoria em vários níveis para ver as conexões teóricas que muitas vezes são apenas implícitas^29^^), (^^30^^), (^^35^.

Pode-se considerar que talvez o mais profícuo uso da interseccionalidade seja como uma estratégia analítica ou disposição para investigar e produzir novos conhecimentos sobre fenômenos sociais^36^. Assim, a busca por aproximar de forma reflexiva a perspectiva intersecional de uma das mais utilizadas técnicas de análise de dados no campo da saúde, em especial no contexto brasileiro, que é a análise temática, deu-se com a intenção de possibilitar análises inovadoras e ampliadas, sensíveis aos marcadores sociais da diferença, problematizando seus impactos nos contextos de pesquisas e reafirmando a necessária implicação destas com a justiça social e a equidade em saúde.
