# Supplementary material for: Errata
Source: Rev Saude Publica. 2025 Jun 27;59:e5serr. [Article in Portuguese] doi: 10.11606/s1518-8787.2025059005728err (PMC12207888; doi:10.11606/s1518-8787.2025059005728err)
Supplement: Related texts

# Correction: Erratum

In the article “At the intersections: operationalizing intersectional thematic analysis in HIV prevention”, DOI http://doi.org/10.11606/s1518-8787.2020054005728, published on the Revista de Saúde Pública. 2024;58: Suppl 1:5s, RSP corrects:

**How to cite (page 1):**

Where it reads:

http://doi.org/10.11606/s1518-8787.2020054005728

It should read:

http://doi.org/10.11606/s1518-8787.2024058005728

**Footnote (pages 1 to 10):**

Where it reads:

http://doi.org/10.11606/s1518-8787.2020054005728

It should read:

http://doi.org/10.11606/s1518-8787.2024058005728
